# Evolution of alternative insect life histories in stochastic seasonal environments

**DOI:** 10.1002/ece3.2310

**Published:** 2016-07-15

**Authors:** Sami M. Kivelä, Panu Välimäki, Karl Gotthard

**Affiliations:** ^1^ Department of ZoologyStockholm UniversitySE‐10691StockholmSweden; ^2^ Department of EcologyUniversity of OuluPO Box 3000FI‐90014OuluFinland

**Keywords:** Bet‐hedging, clinal variation, geometric mean fitness, life cycle, phenotypic plasticity, voltinism

## Abstract

Deterministic seasonality can explain the evolution of alternative life history phenotypes (i.e., life history polyphenism) expressed in different generations emerging within the same year. However, the influence of stochastic variation on the expression of such life history polyphenisms in seasonal environments is insufficiently understood. Here, we use insects as a model and explore (1) the effects of stochastic variation in seasonality and (2) the life cycle on the degree of life history differentiation among the alternative developmental pathways of direct development and diapause (overwintering), and (3) the evolution of phenology. With numerical simulation, we determine the values of development (growth) time, growth rate, body size, reproductive effort, adult life span, and fecundity in both the overwintering and directly developing generations that maximize geometric mean fitness. The results suggest that natural selection favors the expression of alternative life histories in the alternative developmental pathways even when there is stochastic variation in seasonality, but that trait differentiation is affected by the developmental stage that overwinters. Increasing environmental unpredictability induced a switch to a bet‐hedging type of life history strategy, which is consistent with general life history theory. Bet‐hedging appeared in our study system as reduced expression of the direct development phenotype, with associated changes in life history phenotypes, because the fitness value of direct development is highly variable in uncertain environments. Our main result is that seasonality itself is a key factor promoting the evolution of seasonally polyphenic life histories but that environmental stochasticity may modulate the expression of life history phenotypes.

## Introduction

The effects of temporal environmental uncertainty on life history evolution have been extensively studied. Life history theory predicts that temporal uncertainty of the environment favors temporal dispersion of reproduction (i.e., iteroparity; Schaffer 1974a; Tuljapurkar and Wiener 2000; Wilbur and Rudolf 2006; but see Orzack and Tuljapurkar 1989) and/or variable or delayed time of maturation (Cohen 1966; Tuljapurkar 1990; Tuljapurkar and Istock 1993; Menu et al. 2000; Tuljapurkar and Wiener 2000; Wilbur and Rudolf 2006; Koons et al. 2008). Entering dormancy (called diapause in insects) is a way of delaying maturation, resulting in “germ banking” (Evans and Dennehy 2005). The dormant part of a population acts as a buffer against environmental uncertainty and facilitates population (or genotype) survival if the nondormant individuals fail to reproduce (Phillippi 1993; Tuljapurkar and Istock 1993; Menu et al. 2000; Gourbière and Menu 2009). Risk spreading by a single genotype expressing different phenotypes reduces fitness variance and/or between‐individual correlations in fitness and is known as evolutionary “bet‐hedging” (Seger and Brockmann 1987; Phillippi and Seger 1989; Starrfelt and Kokko 2012). Diversified bet‐hedging strategies (sensu Starrfelt and Kokko 2012) arise via environmental effects so that alternative phenotypes are expressed with certain probabilities to environmental cues, a phenomenon also known as adaptive “coin flipping” (Cooper and Kaplan 1982) and “stochastic polyphenism” (Walker 1986).

In seasonal environments, environmental cues indicating the phase of the predictable seasonal cycle, such as photoperiod or temperature, may affect the probabilities of expressing alternative dormancy phenotypes (e.g., Tauber et al. 1986; Danks 1987). A sharp switch from maturation to dormancy is predicted at a certain point of the favorable season (hereafter referred to as “season”) in deterministic environments (Cohen 1970; Taylor 1980, 1986a,b). Environmental uncertainty that characterizes even seasonal environments would, in turn, favor a gradual shift so that both dormant and nondormant phenotypes are expressed over a large part of the season (Seger and Brockmann 1987; see also Halkett et al. 2004; Välimäki et al. 2008). Seasonal change from maturation to dormancy represents phenotypic plasticity to environmental cues predicting future conditions (“predictive plasticity” sensu Cooper and Kaplan 1982). The phenomenon includes, however, a component of bet‐hedging (“coin flipping” or “stochastic polyphenism”) in response to the uncertainty in the timing of the end of the season when temporal overlap in the expression of dormant and nondormant phenotypes prevails. Predictive plasticity is adaptive because the expression of alternative phenotypes matches those environmental states where they result in high fitness (see Moran 1992). Examples of animal polyphenisms arising via predictive plasticity include taxonomically widespread morphs with or without antipredator defences (Lively 1986; Harvell 1990; Brönmark and Miner 1992; McCollum and Van Buskirk 1996) and a broad array of seasonal polyphenisms among insects (West‐Eberhard 2003; Simpson et al. 2011).

Seasonally polyphenic insects are excellent models for studying how temporal environmental uncertainty affects life history evolution in species expressing predictive plasticity as long as taxon‐specific seasonal adaptations are rigorously taken into account. Diapause (i.e., dormancy in insects) only occurs in a species‐specific life stage, the diapause stage varying from egg to adult (or anything in between) among species (Tauber et al. 1986; Danks 1987). Many insects take advantage of long seasons by completing two or more generations within a single season (e.g., Blanckenhorn and Fairbairn 1995; Välimäki et al. 2008; Aalberg Haugen and Gotthard 2015). This variation in voltinism (the number of generations emerging within a season) is a consequence of alternative developmental pathways of diapause and direct development. The developmental switch (Nijhout 2003) between the pathways is mainly induced by photoperiod as that offers a reliable environmental cue of the remaining time until the onset of the adverse season (Tauber et al. 1986; Danks 1987). The induction of a developmental pathway has life history correlates, commonly resulting in the expression of discrete alternative life history phenotypes at different phases of the seasonal cycle (i.e., seasonal polyphenism; reviewed in Kivelä et al. 2013). In several moth species, for example, individuals developing late in the season attain a larger body size than the ones developing early in the season (Teder et al. 2010).

Life history differences between the alternative developmental pathways are predicted to arise because, in age‐structured populations in deterministic environments, direct development into a reproductive adult generally selects for faster development and shorter reproductive life span compared to diapause (Kivelä et al. 2013). Empirical data are largely consistent with these predictions (Spence 1989; Wiklund et al. 1991; Blanckenhorn 1994; Blanckenhorn and Fairbairn 1995; Kivelä et al. 2012; Aalberg Haugen and Gotthard 2015). Moreover, theory predicts direct development to be associated with a smaller (or equal) body size and a lower fecundity than diapause in deterministic environments (Kivelä et al. 2013). Empirical data are largely consistent with these predictions too (Blanckenhorn 1994; Blanckenhorn and Fairbairn 1995; Teder et al. 2010; Aalberg Haugen and Gotthard 2015; but see Wiklund et al. 1991; Kivelä et al. 2012). Despite good consistency between theory and observations, further development of the theory is warranted, given the ubiquity of stochastic variation in seasonal environments. Earlier theoretical analyses of insect life history evolution in relation to seasonality have generally assumed a deterministic environment and either concentrated on the evolution of local adaptations (Roff 1980; Iwasa et al. 1994; Kivelä et al. 2009, 2013) or within‐season variation in the expression of alternative life histories (Abrams et al. 1996; Gotthard et al. 2007; Kivelä et al. 2013). However, stochasticity effects on insect life history evolution and polyphenic expression of life history phenotypes have largely been neglected (but see Roff 1983; Halkett et al. 2004). This is surprising given the diversity and ecological significance of insects (Stork 2003), the effect of climate variability on biological processes where insects are involved (e.g., the parasitism rate of caterpillars: Stireman et al. 2005), the studies on various stochasticity and seasonality effects on life history evolution and plasticity in other arthropods (Arbačiauskas 2001; Varpe et al. 2009; Barbosa et al. 2015), and the diversity of alternative developmental pathways and consequent alternative phenotypes in the animal kingdom (Lively 1986; Harvell 1990; Brönmark and Miner 1992; McCollum and Van Buskirk 1996; Radwan et al. 2002; West‐Eberhard 2003; Bonte et al. 2014), many of which arise via predictive plasticity.

Stochastic variation in season length, particularly the uncertainty in the endpoint of the season, may favor predictive plasticity in voltinism and development time (Roff 1983). The last generation(s) emerging within a season may respond to cues indicating the forthcoming adverse season by entering diapause instead of direct development (plasticity in voltinism) or developing faster (development time plasticity), or by a combination of these two responses (Roff 1983). However, Roff's (1983) analysis did neither consider alternative developmental pathways and associated life history variation nor variation in adult life histories. He also assumed reproduction to be a point event in time (i.e., extreme semelparity), resulting in complete synchrony of the life cycle across generations and no age structure in the adult population. These assumptions preclude the possibility for partial generations where only the earliest offspring produced by a particular generation enter direct development and thus undermine applicability of the model for predicting life history evolution. Shifts in voltinism are commonly associated with the emergence of partial generations in nature (e.g., Blanckenhorn and Fairbairn 1995; Välimäki et al. 2008; Aalberg Haugen and Gotthard 2015), so age‐structured life history models are needed to derive realistic predictions. Moreover, a gradual response to uncertainty is predicted for aphids in the form of stochastic polyphenism (“coin flipping”). The production of sexual offspring gradually increases toward the end of the season at the expense of investment in asexual offspring. This decreases the growth rate of a clone, but enhances survival over winter via sexually produced diapause eggs (Halkett et al. 2004). A general implication of stochastic polyphenism in aphids would be that stochasticity may affect the expression of alternative developmental pathways through the season when the probability of surviving the adverse season varies between the alternative phenotypes.

In this article, we analyze how unpredictability in seasonality affects the evolution of adaptive life history divergence between the alternative developmental pathways via predictive plasticity. In deterministic seasonal environments, the shortness of time available for the directly developing generation to complete its life cycle selects for unequal allocation of time between the directly developing and diapause generations, which favors life history divergence between the developmental pathways (Kivelä et al. 2013). When the length and beginning date of the season vary among years, time allocation between the two generations, as well as the respective generations' contribution to fitness, may vary greatly. This may change the selection pressures compared to those predicted for a deterministic environment (see also Benton and Grant 1996). Stochasticity in season length increases the variance in direct generation fitness contribution and may favor the evolution of a bet‐hedging strategy where only a proportion of offspring of the diapause generation enter direct development (i.e., partially bivoltine [two generations per season] phenology; cf. Seger and Brockmann 1987; Halkett et al. 2004; Kivelä et al. 2013). Such a life history strategy is likely to also influence the differential expression of life history traits among the alternative developmental pathways. Even if bet‐hedging in diapause induction (Seger and Brockmann 1987) would be a general response to environmental uncertainty, it remains unclear whether such a response would also favor the expression of alternative life histories (sensu Moran 1992) between diapausing and directly developing individuals.

Here, we use climate data to assess realistic stochastic variation associated with the predictable seasonal cycle and incorporate that stochasticity into our recent modeling framework (Kivelä et al. 2013) that proved powerful in predicting alternative life histories in deterministic environments. This allows us to explore the evolution of alternative life history phenotypes in seasonal environments including a stochastic component. This extension of the analysis is crucial for our understanding of the evolution of insect life histories in natural selection regimes that typically show such environmental stochasticity. To explore whether environmental uncertainty favors divergent life history phenotypes between diapause and direct development pathways, we analyze (1) life history differentiation between the pathways (i.e., the evolution of seasonal life history polyphenism) in relation to two realistic axes of environmental uncertainty: unpredictability in season length and in thermal conditions within a season. We also explore (2) the effect of life cycle (the overwintering developmental stage: egg vs. pupa/adult) on life history differentiation between the diapause and direct development phenotypes in bivoltine populations. This is because specific life history events should occur at the right time of the seasonal cycle, and the timing of those events depends both on the overwintering developmental stage and the life histories expressed under diapause and direct development (Roff 1983, 2002; Iwasa et al. 1994; Kivelä et al. 2013). Finally, we analyze (3) the evolution of voltinism and life history divergence among populations along a gradient of changing mean season length. For simplicity, we assume density‐independent selection throughout.

## Materials and Methods

We first analyze climate data in order to realistically model the stochastic component in seasonality. Secondly, we further develop our life history model (Kivelä et al. 2013) to facilitate the analysis of life history evolution under seasonal but stochastic environments. Thirdly, we analyze the evolution of alternative life history phenotypes associated with the alternative developmental pathways of diapause and direct development in a hypothetical insect under climatic selection regimes as characterized by the climate data.

### Climate data

To investigate the associations among mean season length, variation in season length and season beginning across years as well as variation of a risk of frosts within a season, climate data (Nationell kartläggning av klimatdata för Sveriges miljöövervakning, PTHBV version 3 2011, framtaget av SMHI med stöd av Naturvårdsverket; available at http://luftweb.smhi.se/, accessed on 9th of October 2013) from 38 locations arbitrarily selected across Sweden (Fig. S1) were analyzed. Data on mean daily temperatures from 1961 to 2012 interpolated on a 4 × 4 km grid were used. The length of the season when mean daily temperature was above 10°C, *T*, was estimated for each year for each location. Using a 10°C threshold in the mean daily temperature in season delimitation seems realistic in the light of estimated minimum temperatures for development in several insects (Dixon et al. 2009). Moreover, the qualitative results (significances of correlations among climate variables; see below) remain unchanged when the threshold is 6–10°C (the only insignificant correlation becomes significant with thresholds above 10°C, while the others remained unaffected). Season was determined to begin on the first date within a year when the mean temperature exceeded 10°C and remained above it at least seven subsequent days. Season was determined to end on the last date when mean temperature exceeded 10°C and was followed by at least 7 days when mean temperature was below 10°C. These definitions closely follow the definition of meteorological summer in Sweden (SMHI, http://www.smhi.se/kunskapsbanken/meteorologi/arstider-1.1082, cited on 22nd of February 2016).

We define a parameter *τ* to describe within‐season risk of a frost. A frost (we refer to frost as a lethally low temperature in general) was assumed to occur on those days when mean temperature was below 10°C during the season. As our aim is to estimate the risk of within‐season frosts within a location with a particular climate (i.e., the selection regime from the biological point of view) and season length varies among years within a location, we first transformed the occurrences of frosts from dates to proportions of season elapsed since the beginning of the season in each year included in the data set. The climatic selection regime is largely determined by the mean season length, and thus, we back‐transformed the above proportions to dates (*t*; days since the beginning of the season), but for a season whose length was the mean season length ($\overset{¯}{T}$; rounded to the nearest integer). Thirdly, we calculated the date‐specific numbers of frost observations for the average season. Dividing these date‐specific numbers of frost observations by the number of studied years (52) gives frost probabilities (*p*_frost_) for each date. Parameter *τ* was estimated by fitting a function

(1) $$p_{\mathrm{frost}}={\left(\frac{t}{\overset{¯}{T}+1}\right)}^{\tau }$$

to these data with the function *nls* in R 3.0.1 (R Core Team 2013) using the “nl2sol” algorithm. Equation (1) means that the frost risk is a monotonically increasing function of time within a season (we required that *τ *> 0), and the risk will reach one on the first day after the termination of the season, following the definition for season end. Even with the constraint of frost risk reaching one the day after season end, the fit to data was reasonably good on the grounds of visual evaluation and residual standard error (mean = 0.149 [95% CI: 0.131–0.166]), justifying our approach. Note that decreasing *τ* means increasing frost risk through the season (Fig. S2).

We ignored early‐season frosts in our analysis because the synchronous beginning of postdiapause development in the diapause generation means that there is strong selection for starting postdiapause development at a time in the year when the frost risk is low. Furthermore, our definitions for the dates of season beginning and end result in a relatively “safe” early season, whereas the end of the season is typically preceded with exaggerated frost risk. Owing to these reasons, late‐season frosts are expected to influence the evolution of seasonal life history polyphenisms.

**Figure 1:**
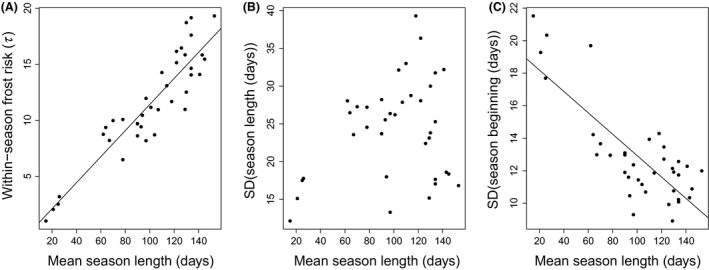
Parameter *τ* that describes within‐season frost risk (A), standard deviation in the length of the favorable season (referred to as season; (B) and standard deviation of season beginning date (C) per location shown according to mean season length. Increasing values of *τ* mean decreasing frost risk. Mean season lengths (mean daily temperature >10°C), values of *τ* and standard deviations of season length and beginning date were estimated from data on daily mean temperatures during 1961–2012 from 38 arbitrary locations in Sweden (see text for details; Fig. S1). The regression equation in (A) is *τ *= 0.116 ×  (mean season length) − 0.223 (regression model *F*_1,36_ = 187, *P *<* *0.0001) and in (C) SD(season beginning) = 19.5 − 0.066 ×  (mean season length) (regression model *F*_1,36_ = 62.3, *P *<* *0.0001).

The aim was to realistically model environmental uncertainty in the life history analysis. Therefore, the relationships among mean season length, variation in season length and season beginning across years, and *τ* were used in the analysis. Parameter *τ* was positively correlated with site‐specific mean season length (Pearson's correlation = 0.916, *t*_36_ = 13.7, *P* < 0.0001; Fig. 1A), indicating that the risk of within‐season frosts increases with decreasing mean season length. Standard deviation of season length was independent of mean season length (Pearson's correlation = 0.261, *t*_36_ = 1.62, *P* = 0.11; Fig. 1B), but standard deviation of season beginning date was negatively correlated with mean season length (Pearson's correlation = −0.796, *t*_36_ = −7.89, *P* < 0.0001; Fig. 1C), indicating that the beginning of the season becomes more predictable with increasing mean season length. To save computing time using shorter season lengths in the life history analysis described below, the observed mean season lengths and standard deviations of season length were rescaled by dividing them by three. Consequently, the rescaled regression equation for *τ* became *τ *= 0.35 × (mean season length) − 0.19 (Fig. 1). The mean season beginning date, *I* (days since 31st December), was determined to be 180 − [scaled mean season length]/2. The rescaled regression equation for the standard deviation of season beginning date became SD (season beginning) = 6.5 − 0.065 × [scaled mean season length] (Fig. 1). Rescaling did not affect the relationships among climate variables, and we used the rescaled regression equations in all subsequent analyses.

### Modeling methods

The model is built on the deterministic model by Kivelä et al. (2013). The model parameters and evolving traits are summarized in Table 1, and details of model derivation are presented in Appendix. Here, we present an outline of the model structure and concentrate on the novel aspects of the model that differ from the deterministic version.

**Table 1:** Input parameters, their input values, and optimized traits of the model. Optimized independent traits are given in boldface, whereas the values of other optimized traits were determined as correlated responses to the independent traits

Parameter type	Parameter	Input value	Description
Input parameter (organismal)	*r*	0, 1	Determines the diapausing developmental stage (0: egg diapause; 1: pupal diapause)
	*B*	3	*B *=* *1/(1 − *g*), *g* being the allometric exponent scaling anabolism to body mass
	*a*	50	Asymptote of fecundity–body mass relationship
	*b*	0.01	Determines the rate at which the asymptote *a* is approached with increasing mass
	*c*_*1*_	2.5	Determines how fecundity increases with increasing reproductive effort
	*c*_*2*_	2	Determines the relationship between survival and reproductive effort
	*t*_pupa_	1	Pupal development time
	*m*_min_	80	Minimum mass for successful metamorphosis
Input parameter (mortality risk)	*M*_ad_	0.15	Daily adult mortality rate
	*M*_juv0_	0.01	Growth rate‐independent component of daily larval mortality rate
	*d*	0.15	Growth rate‐dependent larval daily mortality rate when growth rate is *c*_d_
	*c*_*d*_	0.05	Growth rate resulting in growth rate‐dependent larval daily mortality rate *d*
	*k*	2	Determines the relationship between larval daily mortality rate and growth rate
	*z*	0.001	Minimum permissible larval survival probability
Input parameter (seasonality)	*μ*_T_	36 and 22–45	Mean season length
	*σ*_T_	0–6	Standard deviation of season length
	*μ*_I_	180 − *μ*_T_/2	Mean season beginning date
	*σ*_I_	6.5 − 0.065 × *μ*_T_	Standard deviation of season beginning date
	*τ*	0.35 × *μ*_T_ − 0.19 (±20%)	Determines the risk of frosts during a season (risk increases with decreasing *τ*)
Optimized traits	D*		Critical date of diapause induction
	*c*_Dp,_ *c*_Di_		Growth rates in the diapause and direct development pathways
	***t***_**larva(Dp),**_ ***t***_**larva(Di)**_		Larval development times in the diapause and direct development pathways
	*m*_pupa(Dp)_, *m*_pupa(Di)_		Pupal masses in the diapause and direct development pathways
	***E***_**Dp,**_ ***E***_**Di**_		Reproductive efforts in the diapause and direct development pathways
	*ω*_Dp_, *ω*_Di_		Adult life spans in the diapause and direct development pathways
	*R*_0(Dp)_, *R*_0(Di)_		Expected life time fecundities in the diapause and direct development pathways

We focus on the evolution of larval development time and adult reproductive effort in the diapause and direct development generations as well as the evolution of photoperiodic threshold affecting diapause induction as these are key traits affecting fitness and voltinism. Moreover, we analyze correlated evolution in growth rate, body size, adult life span, and fecundity. The R‐code of the model is available as electronic supplementary appendices (Appendices S1 and S2).

**Figure 2:**
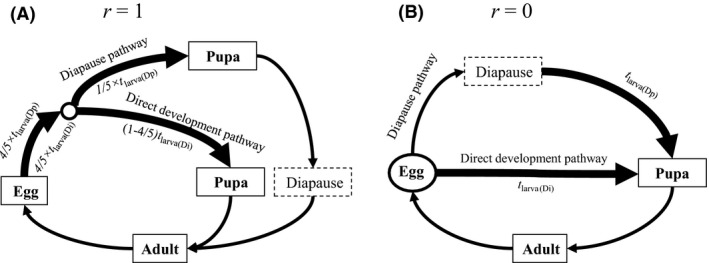
Life cycles with pupal diapause (A) and egg diapause (B) used in the analyses. Arrows depict transitions between different life stages (the durations of those transitions are indicated for larval development) and alternative developmental pathways (indicated by text along the arrows) for juvenile development. Larval development is depicted with thick arrows. The life stage indicated by a circle is where induction of diapause (or direct development) takes place. This sensitive stage for diapause induction is after 4/5 of prediapause juvenile development, whose duration is 4/5 × *rt*_larva(Dp)_ for the diapause pathway and 4/5 × *rt*_larva(Di)_ for the direct development pathway, *r* determining the diapausing developmental stage (*r *=* *1: pupal diapause; *r *=* *0: egg diapause). Individuals following the direct development pathway have uninterrupted juvenile development of duration *t*_larva(Di)_, while individuals following the diapause pathway arrest their development and enter diapause. Diapausing individuals complete postdiapause development of duration 0 and *t*_larva(Dp)_ with pupal and egg diapause, respectively, in the beginning of the favorable season in the following year, after which they become reproductive adults. Note that *r *=* *1 can also be interpreted as adult diapause in this analysis.

The diapausing (i.e., overwintering) developmental stage is the stage occurring after a proportion *r* of the larval development time (duration of larval growth period), *t*_larva_. Hence, the diapausing stage is the pupa (or the adult) if *r *=* *1, the egg if *r *=* *0, and the larva if 0 < *r *<* *1. The life cycles with *r *=* *1 and *r *=* *0 are illustrated in Figure 2.

Postdiapause development of duration (1 − *r*)*t*_larva_ (rounded to the nearest integer) is completed at the beginning of the season. Adults then reproduce. The induction of a developmental pathway in the offspring cohorts takes place after development has continued for 4/5 × *rt*_larva_ (rounded to the nearest integer) days as diapause induction generally takes place shortly before the developmental stage where the diapause is effectuated (Danks 1987; Friberg et al. 2011). If offspring reach the sensitive stage for diapause induction before the critical date of diapause induction, D* (days since 31st December), direct development is induced. Otherwise diapause is induced. We allow at most two generations to emerge within a season, so the offspring of the possible direct generation will invariably enter diapause and express diapause pathway trait values. Only individuals that follow the diapause pathway and complete the prediapause development of duration *rt*_larva_ (rounded to the nearest integer) before the end of the season will survive winter. Annual fitness is calculated as the number of surviving descendants produced within a season, *R* (annual rate of increase; assuming no winter mortality), which is appropriate for temperate insects when only a few generations are completed within a season (Roff 1980, 1983, 2008; Kivelä et al. 2013). Long‐term fitness is defined as the geometric mean, *G*, of the annual rates of increase, as it is an appropriate fitness currency in stochastic environments without density‐dependent selection (Dempster 1955; Gillespie 1977; Yoshimura and Jansen 1996; Roff 2002, 2008). Across *n* years (see “Analyses” section for deriving the years to be analyzed), *G* can be calculated as

(2) $$G=e^{\frac{1}{n}\sum_{i=1}^{n}\ln\left(R_{i}\right)}$$

where *R*_*i*_ is the rate of increase in year *i*.

We refer the diapause pathway to the subscript “Dp” while the subscript “Di” stands for the direct development pathway. The procedure to find the developmental pathway‐specific values of larval development time (*t*_larva(Dp)_, *t*_larva(Di)_), growth rate (*c*_Dp_, *c*_Di_), pupal mass (*m*_pupa(Dp)_, *m*_pupa(Di)_), reproductive effort (*E*_Dp_, *E*_Di_), adult life span (*ω*_Dp_, *ω*_Di_), and expected lifetime fecundity (*R*_0(Dp)_, *R*_0(Di)_) that maximize geometric mean fitness largely follows the procedure used by Kivelä et al. (2013). Larval development time (*t*_larva(•)_) and adult reproductive effort (*E*_*•*_) were considered independent traits as they are key traits affecting fitness and voltinism. Body size (*m*_pupa(•)_), adult life span (*ω*_*•*_), and expected lifetime fecundity (*R*_*0*(•)_) are determined by larval development time (*t*_larva(•)_), growth rate (*c*_*•*_), and reproductive effort (*E*_*•*_). Growth rate optimization follows the procedure by Kivelä et al. (2013) and is explained in the Appendix.

Geometric mean fitness maximizing life history was found by three‐stage nested numerical optimization procedure that is a slightly modified version from the procedure used by Kivelä et al. (2013). Phenology is determined by the duration of postdiapause development, (1 − *r*)*t*_larva(Dp)_, larval development time in the direct development pathway, *t*_larva(Di)_, the beginning date of the season, *I* (days since 31st December), and the critical date of diapause induction, D* (days since 31st December). In the first stage of the analysis (1), season length was set to the mean season length, *μ*_T_, and season beginning to the mean season beginning date, *μ*_I_ (rounded to the nearest integer). Given values for *t*_larva(Dp)_, *E*_Dp_, *r*,* μ*_T_, *μ*_I*,*_ and D*, the date of emergence for a diapause generation female, its expected age‐specific fecundities, and the consequent sizes and hatching dates of offspring cohorts were derived. Then, the iterative procedure by Kivelä et al. (2013) was used to solve *t*_larva(Di)_ whenever a direct generation could emerge: Firstly (i), the shortest possible *t*_larva(Di)_ was used in determining the cohorts entering direct development and then (ii) direct generation *t*_larva(Di)_ and *E*_Di_ were optimized independently of the initially assigned *t*_larva(Di)_ by calculating *R*_0(Di)_ in each combination of the analyzed values of *t*_larva(Di)_ and *E*_Di_, and finding the combination of trait values (*t*_larva(Di)_ and *E*_Di_) that maximized *R*_0(Di)_. Then (iii), if the optimization of *t*_larva(Di)_ and *E*_Di_ in (ii) resulted in a different value of *t*_larva(Di)_ as the one used in determining the directly developing cohorts in (i), the step (i) was repeated with 1 day longer *t*_larva(Di)_ determining the directly developing cohorts, followed by (ii) the independent optimization of *t*_larva(Di)_ and *E*_Di_. This procedure was repeated until the optimization of *t*_larva(Di)_ and *E*_Di_ in step (ii) resulted in the same *t*_larva(Di)_ as the one used in determining directly developing cohorts in step (i).

Next, geometric mean fitness was calculated for the life history under investigation, that is, for the life history whose direct generation trait values were determined as described above. The trait values (*t*_larva(Di)_, *c*_Di_, *m*_pupa(Di)_, *E*_Di_, *ω*_Di_ and *R*_0(Di)_) found with the above iterative procedure were used, given *t*_larva(Dp)_, *E*_Dp_, *r*,* μ*_T_, *μ*_I_, *τ,* and D*, and the annual rates of increase, *R*, were calculated for this life history (note that D* is also a life history trait) for each random realization of season length and season beginning date (rounded to the nearest integer) that were derived from the assumed distributions (season length, *T ˜ N(μ*_*T*_, *σ*_*T*_ *)*; season beginning, *I ˜ N(μ*_*I*_, *σ*_*I*_ *)*). For calculation of *R*, the sizes of surviving cohorts of diapausing descendants entering the overwintering population were weighed by the cohort‐specific probabilities to survive within‐season frosts. This was performed so that, for each random realization of season length, *T'*, the sizes of diapause generation cohorts that managed to complete their prediapause development on day *t* (*t *≤ *T'*) were multiplied by $\prod_{y=1}^{t}\left(1-{\left(y/\left(T^{\prime }+1\right)\right)}^{\tau }\right)$ (see equation (1)), where index *y* denotes to the number of days since the beginning of the season. Then, the geometric mean fitness, *G* (equation (2)), was calculated for the life history in question over the analyzed seasons.

In the second stage of the analysis (2), the above determination of the geometric mean fitness of a life history (stage 1) was repeated at each combination of the analyzed values of *t*_larva(Dp)_ and *E*_Dp_ to find the values of *t*_larva(Dp)_ and *E*_Dp_ (and associated *t*_larva(Di)_ and *E*_Di_; see the above procedure for finding *t*_larva(Di)_ and *E*_Di_ associated with particular *t*_larva(Dp)_ and *E*_Dp_) that maximized *G* with the given values of D*,* r*,* μ*_*T*_, *μ*_*I*._ and *τ*. In the third stage (3), the value of D* that maximized *G*, with the given values of *r*,* μ*_T*,*_ and *μ*_I*,*_ was found by repeating the stages (1) and (2) with different values of D*. The earliest analyzed D* induced diapause in all cohorts, and testing later positions of D* was continued only until the maximum of *G* was found, which corresponds to the global optimum for D* because *G* is a unimodal or an asymptotic function of D* (Kivelä et al. 2013). The life history found at this final (3) stage maximized geometric mean fitness in the given selection regime (i.e., particular *μ*_T_, *σ*_T_, *μ*_I_, *σ*_I_, *τ*,* M*_ad_, *M*_juv0_, *k*,* d,* and *c*_d_).

### Analyses

Life history differentiation between the alternative developmental pathways was analyzed in relation to two axes of environmental unpredictability: variation in season length among years (*σ*_T_) and the risk of low lethal temperatures (referred to as frosts) within a season (*τ*). Variation in season beginning date (*σ*_I_) was strongly correlated with mean season length (Fig. 1C), which is why we did not add it as a third axis of environmental unpredictability in the analyses but modeled it to depend on mean season length as *σ*_I_ = 6.5–0.065 × *μ*_T_ (see the “Climate data” section). Hence, we maximized geometric mean fitness with different values of *σ*_T_ and *τ* (and correlated values of *σ*_I_), in the analyses described below, and present life history variation in relation to *σ*_T_ and *τ*.

We analyzed the evolution of alternative life histories under strong time constraints for bivoltine phenology. Preliminary analyses showed that the shortest season lengths where bivoltinism evolved (*σ*_T_ = 0) were 34 and 33 days with pupal and egg diapause, respectively. [Note that the transitions from univoltine to bivoltine phenology occurred at somewhat shorter season lengths in the deterministic analyses by Kivelä et al. (2013). This is because we included the effect of within‐season frosts and variation in season beginning date to the current analysis even when *σ*_T_ was set to 0.] Therefore, we conduct the analyses at a mean season length of 36 days. We repeated the analysis for both the pupal (*r *=* *1) and egg (*r *=* *0) ends of the continuum of possible overwintering developmental stages to analyze the limits within which the life cycle affects the evolution of alternative life histories.

The analyzed values of *σ*_T_ were those from 0 to 6 with an increment of 0.5 (13 values in total). The analyzed values of *τ* included 11 equally spaced values from 0.8 ×  (0.35 × *μ*_T_ − 0.19) to 1.2 × (0.35 × *μ*_T_ − 0.19). Geometric mean fitness was maximized over 200 random realizations of season length (*T*) and season beginning date (*I*), both derived from the particular normal distributions assumed for them (i.e., *T ˜ N(μ*_T_, *σ*_T_ *)*,* I ˜ N(*180 − *μ*_T_/2, 6.5–0.065 × *μ*_T_ *)*; see the “Climate data” section for derivation of *μ*_I_ and *σ*_I_). In all combinations of the analyzed values of *σ*_T_ and *τ*, given values of *μ*_T_ and *r*, the analyzed season lengths were derived using the same seed of the pseudo random number generator to minimize random noise effects on the comparisons within the *σ*_T_ − *τ* grid under investigation.

Finally, we analyzed the effect of a change between univoltine and bivoltine phenologies on life history evolution by deriving predictions for clinal variation in the analyzed traits in relation to mean season length. In this analysis, we set *τ *= 0.35 × *μ*_T_ − 0.19 and *σ*_I_ = 6.5–0.065 × *μ*_T_. Mean season lengths from 22 to 45 days were analyzed (the change in voltinism takes place approximately in the middle of this gradient; see above) with pupal and egg diapause using the *σ*_T_ values of 0 and 4 days. As earlier, geometric mean fitness was maximized over 200 random realizations of season length and season beginning date that always were derived with the same seed for the pseudo random number generator to minimize noise on comparisons among different *μ*_T_, and among different values of *σ*_T_.

## Results

**Figure 3:**
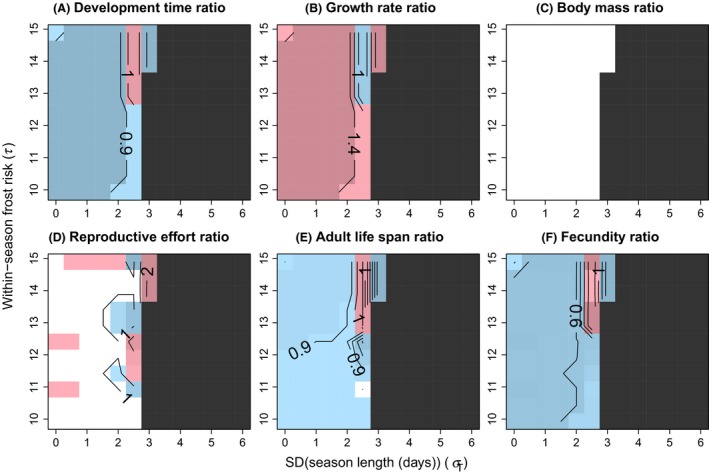
Contour diagrams of relative life history differences between the direct development and the diapause pathway with pupal diapause. Trait value in the direct development pathway is divided by the trait value in the diapause pathway. The ratio is shown for larval development time (A), larval growth rate (B), pupal mass (C), reproductive effort (D), adult life span (E), and lifetime fecundity (F) in relation to standard deviation of season length (*σ*_*T*_) and within‐season risk of frosts (*τ*; risk increases with decreasing value of *τ*). Ratios less than one (i.e., trait value in the direct development pathway < trait value in the diapause pathway) are indicated by blue and ratios higher than one (i.e., trait value in the direct development pathway > trait value in the diapause pathway) by red. Darker color indicates increasing differentiation between the developmental pathways (note that a particular darkness of blue or red indicates different trait differentiation in different panels), and a ratio of one (i.e., equality of life histories) is indicated by white color. Dark gray indicates a region where a direct generation does not emerge. Note that the coarse pattern arises because the model included both continuous and discrete traits. The maximized variable (geometric mean fitness) is continuous and varies smoothly across the analyzed selection regimes (Fig. A1), but whenever a value of an underlying discrete trait changes, there are associated changes in all the other analyzed traits as well, which makes variation in the relative trait values presented here coarse and partially obscures general patterns. Parameter values were: *c*_1_ **= 2.5, *c*_2_ = 2, *a *=* *50, *b *=* *0.01, *c*_d_ = 0.05, *k *=* *2, *z *=* *0.001, *B *=* *3, *m*_min_ = 80, *t*_pupa_ = 1, *d *=* *0.15, *M*_juv0_ = 0.01, *M*_ad_ = 0.15, *r *=* *1, *μ*_T_ = 36, *μ*_I_ = 162, and *σ*_I_ **= 4.16.

**Figure 4:**
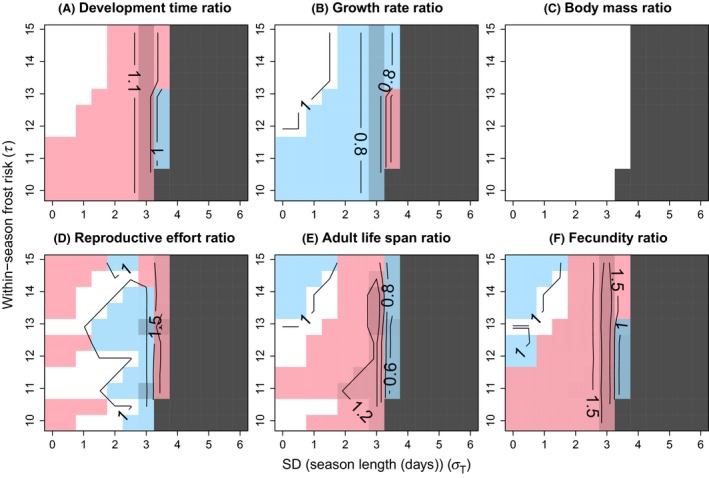
Contour diagrams of relative life history differences between the direct development and the diapause pathway with egg diapause. Trait value in the direct development pathway is divided by the trait value in the diapause pathway. The ratio is shown for larval development time (A), larval growth rate (B), pupal mass (C), reproductive effort (D), adult life span (E), and lifetime fecundity (F) in relation to standard deviation of season length (*σ*_T_) and within‐season risk of frosts (*τ*; risk increases with decreasing value of *τ*). See Figure 3 for explanation of the colors and the coarse pattern of variation. Parameter values were as follows: *c*_1_ **= 2.5, *c*_2_ = 2, *a *=* *50, *b *=* *0.01, *c*_d_ = 0.05, *k *=* *2, *z *=* *0.001, *B *=* *3, *m*_min_ = 80, *t*_pupa_ = 1, *d *=* *0.15, *M*_juv0_ = 0.01, *M*_ad_ = 0.15, *r *=* *0, *μ*_T_ = 36, *μ*_I_ = 162, and *σ*_I_ **= 4.16.

**Figure 5:**
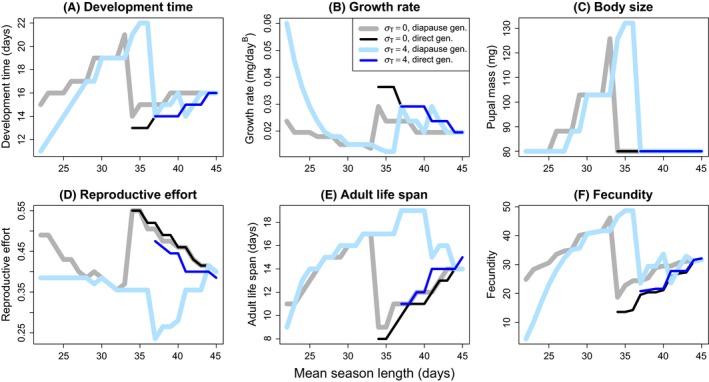
Clinal variation in larval development time (duration of growth; A), growth rate (B), pupal mass (C), reproductive effort (D), adult life span (E), and lifetime fecundity (F) in the diapause (thick lines) and direct development (thin lines) pathways in relation to mean length of the favorable season (*μ*_T_) with pupal diapause. The predictions are presented for two standard deviations of season length: *σ*_T_ **= 0 (gray and black lines) and *σ*_T_ **= 4 days (light blue and dark blue lines). Note that direct development is only expressed (i.e., phenology is bivoltine) when mean season length is relatively long. Parameter values were as follows: *c*_1_ **= 2.5, *c*_2_ = 2, *a *=* *50, *b *=* *0.01, *c*_d_ = 0.05, *k *=* *2, *z *=* *0.001, *B *=* *3, *m*_min_ = 80, *t*_pupa_ = 1, *d *=* *0.15, *M*_juv0_ = 0.01, *M*_ad_ = 0.15, *τ *= 0.35 × *μ*_T_ − 0.19, *μ*_I_ = 180 − *μ*_T_/2, *σ*_I_ **= 6.5–0.065 × *μ*_T*,*_ and *r *=* *1.

**Figure 6:**
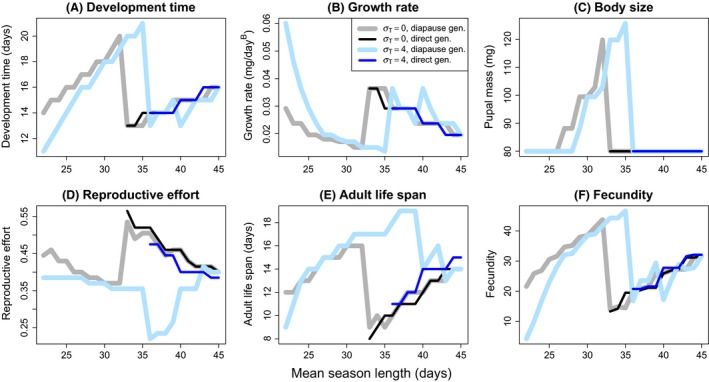
Clinal variation in larval development time (duration of growth; A), growth rate (B), pupal mass (C), reproductive effort (D), adult life span (E), and lifetime fecundity (F) in the diapause (thick lines) and direct development (thin lines) pathways in relation to mean length of the favorable season (*μ*_T_) with egg diapause. See Figure 5 for explanation of line types and colors. Parameter values were as follows: *c*_1_ **= 2.5, *c*_2_ = 2, *a *=* *50, *b *=* *0.01, *c*_d_ = 0.05, *k *=* *2, *z *=* *0.001, *B *=* *3, *m*_min_ = 80, *t*_pupa_ = 1, *d *=* *0.15, *M*_juv0_ = 0.01, *M*_ad_ = 0.15, *τ *= 0.35 × *μ*_T_ − 0.19, *μ*_I_ = 180 − *μ*_T_/2, *σ*_I_ **= 6.5–0.065 × *μ*_T*,*_ and *r *=* *0.

Divergent life history phenotypes were generally predicted to evolve in the diapause and direct development pathways in the analyzed selection regimes whenever bivoltine phenology evolved (Figs. 3, 4). Increasing interyear standard deviation of season length induced a switch from bivoltine to univoltine phenology (Figs. 3, 4), indicating that environmental variation constrains the evolution of bivoltinism. On the other hand, the change between univoltine and bivoltine phenologies moved toward longer mean season lengths when the variation in season length increased (Figs. 5, 6). The risk of within‐season frosts had a minor effect on the evolution of phenology; the switch from bivoltine to univoltine phenology slightly moved toward higher variation in season length with decreasing within‐season frost risk (decreasing frost risk means increasing *τ*; Figs. 3, 4).

There was a clear effect of life cycle (parameter *r*) on the predicted trait differentiation. With pupal (or adult) diapause (*r *=* *1), shorter larval development time (growth duration), higher growth rate, shorter adult life span, and lower lifetime fecundity were generally predicted under direct development than diapause (Fig. 3). The predicted direction of trait differentiation was, however, mainly in the opposite direction with egg diapause (*r *=* *0) as direct development was predicted to associate with longer larval development time, lower growth rate, longer adult life span, and higher lifetime fecundity than diapause in a major part of the parameter space where bivoltinism was predicted to emerge (Fig. 4). Moreover, a larger part of parameter space produced equal trait values of the two alternative developmental pathways when diapause was in the egg stage compared with pupal diapause (compare Fig. 4 to Fig. 3). No differentiation in body size was predicted with either life cycle (Figs. 3C, 4C) as the strong time constraints allowed only the minimum size for successful metamorphosis to be attained in both developmental pathways. The predictions for reproductive effort were qualitatively very similar both with pupal and egg diapause (Figs. 3D, 4D). Equal reproductive efforts were predicted in a large part of the analyzed parameter space, and where trait differentiation was predicted, it was about equally often in both directions. Yet, differentiation of reproductive effort between the pathways was more often predicted with egg diapause.

Clinal variation in relation to mean season length was predicted in each analyzed trait, with a major effect of changing phenology (Figs. 5, 6). We derived predictions for clinal variation also with no variation in season length among years (*σ*_T_ = 0) for a baseline. Yet, this baseline is not completely identical with the one predicted in a deterministic environment (see Kivelä et al. 2013), because within‐season frost risk and variation in season beginning date were still included in the present analysis. The predicted clinal variation in our present baseline analysis (*σ*_T_ = 0) was, however, very similar to that predicted in completely deterministic environments; development time and adult life span lengthen, growth rate and reproductive effort decrease, and body size and expected lifetime fecundity increase with increasing season length as long as voltinism remains constant (body size remained constant under bivoltine phenology within the analyzed range of mean season length), an abrupt change taking place in each trait at the shift from a univoltine to a bivoltine phenology (Figs. 5, 6).

Interyear variation in season length affected the predicted life history clines in relation to mean season length. Increasing standard deviation of season length (*σ*_T_) from 0 to 4 days moved the change from univoltine to bivoltine phenology to a longer mean season length (Figs. 5, 6) and changed the clines predicted for reproductive effort and adult life span qualitatively. Reproductive effort abruptly increased at the change from univoltine to bivoltine phenology with *σ*_T_ = 0, reproductive effort being very similar in both diapause and direct development pathways. Yet, *σ*_T_ = 4 produced a cline where reproductive effort abruptly decreased in the diapause pathway at changing phenology, whereas reproductive effort in the direct development pathway was much higher and close to values predicted with *σ*_T_ = 0 (Figs. 5, 6). The reproductive efforts in the diapause and direct development pathways gradually converged with increasing mean season length. In adult life span, there was no abrupt change in the diapause pathway trait value at the change in phenology (with *σ*_T_ = 4), but it continued to lengthen toward increasing mean season length until it sharply shortened at a mean season length where the developmental pathway‐specific reproductive efforts converged. However, adult life span in the direct development pathway was much shorter than in the diapause pathway, and close to that predicted with *σ*_T_ = 0, immediately after the change to a bivoltine phenology (Figs. 5, 6). With increasing mean season length, adult life span in the direct development pathway lengthened and reached approximately the same level as in the diapause pathway after the sharp decrease in the trait value of the diapause pathway (Figs. 5, 6).

The predicted clinal variation showed a similar life cycle effect on the direction of trait differentiation between the alternative developmental pathways as the more comprehensive analysis conducted at a mean season length of 36 days; the direction of development time, growth rate, and fecundity differentiation between the alternative developmental pathways tending to reverse with egg diapause compared to that predicted with pupal (or adult) diapause (compare Fig. 5 to Fig. 6).

## Discussion

Our analysis assuming density‐independent selection in stochastic seasonal environments predicted qualitative adaptive trait differentiation between diapause and direct development pathways (i.e., relative trait values) in organisms such as insects that may complete two generations within a season. The analysis included stochasticity in season length as well as the occurrence of lethal cold spells during the season. The predicted life history differentiation between the alternative developmental pathways (aim 1; cf. Introduction) was generally very similar to that predicted to evolve in deterministic seasonal environments (Kivelä et al. 2013), emphasizing the significance of seasonality as such for the evolution of alternative life history phenotypes via predictive plasticity (Cooper and Kaplan 1982). The predictions are also generally consistent with empirical data (reviewed in Kivelä et al. 2013). Nevertheless, there were some important differences between the predictions derived for deterministic (Kivelä et al. 2013) and stochastically varying seasonal environments (this study). The effect of overwintering developmental stage on the direction of life history divergence between the alternative developmental pathways (aim 2) becomes evident only under environmental stochasticity, and increasing uncertainty in season length decreases investment in the directly developing generation. Decreasing investment in the high risk–high gain direct generation with increasing environmental unpredictability represents bet‐hedging and is in accordance with earlier work predicting increasing expression of dormancy or prolonged dormancy in increasingly stochastic environments (Cohen 1966; Tuljapurkar 1990; Tuljapurkar and Istock 1993; Menu et al. 2000; Tuljapurkar and Wiener 2000; Halkett et al. 2004; Wilbur and Rudolf 2006; Koons et al. 2008; Rajon et al. 2014). Bet‐hedging is also reflected in the predicted clinal variation in life histories (aim 3).

The present predictions concerning trait differentiation with pupal (or adult) diapause parallel those derived for deterministic environments (Kivelä et al. 2013). Direct development is associated with a relatively short development time, high growth rate, short adult life span, and low lifetime fecundity. These differences between the developmental pathways were, however, predicted to reverse or disappear with egg diapause. A qualitatively similar reversal of life history differentiation between the alternative developmental pathways was found when the analysis was repeated at a mean season length of 50 days where time constraints for bivoltine phenology are, on average, relaxed (Appendix S3). The qualitative similarity of results obtained at the two mean season lengths indicates that the result is not specific to the case of strong time constraints for bivoltine phenology where we focused on. The life cycle dependency of trait differentiation found here contrasts with some of the predictions concerning deterministic environments (Kivelä et al. 2013), but agrees with the ones on development time (Iwasa et al. 1994; Roff 2002; see also Appendix S3).

**Figure 7:**
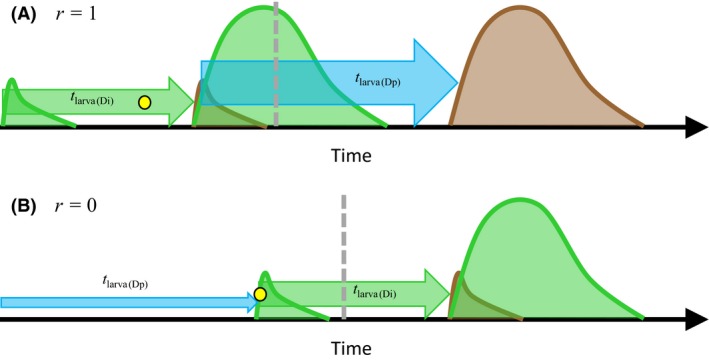
A schematic illustration of the timing of life history events with pupal (A) and egg (B) diapause under bivoltine phenology. The green distributions illustrate reproduction (number of eggs) in the two generations emerging within the season. The brown distributions illustrate pupation (number of pupae) in the offspring generations. The heights of the distributions are not exactly in a scale but illustrate the size difference between the generations. Larval development is depicted with green (direct development generation; duration *t*_larva(Di)_) and blue (diapause generation; duration *t*_larva(Dp)_) arrows, the heights of the arrows indicating the sizes of the generations (not in scale, but illustrate the rank order between generations). The yellow dots indicate the sensitive developmental stage for diapause induction, and the vertical gray dashed line the time when the critical photoperiod of diapause induction occurs. A single female was assumed in the beginning of the season. For the purpose of illustration, pupal development time was assumed to be zero. Note that reproduction extends over a longer time period in the second generation than in the first one because adult emergence is asynchronous in the second generation but synchronous in the first one.

Reversed trait differentiation for juvenile development with egg diapause is understandable in relation to time constraints and the timing of the expression of the diapause and direct development phenotypes (illustrated in Fig. 7). With pupal or adult diapause, the first larval generation emerging within a season expresses the direct development phenotype when phenology is bivoltine. However, with egg diapause, the season's first larval generation expresses the diapause phenotype independently of phenology. If there is selection for bivoltinism, the season's first larval generation will experience time–stress because the development time in the first larval generation determines whether direct development can be induced (i.e., time before the photoperiodic switch to diapause; Fig. 7). This selects for a short larval period in the first larval generation emerging within a season. Note that direct development will be induced in the egg stage with egg diapause, which is why the development time in the first larval generation mainly determines whether the diapause generation adults manage to lay their eggs before photoperiodic switch to diapause. On the other hand, asynchronous reproduction in the direct generation, which is a consequence of age structure in the adult stage, may select for fast development in the direct development pathway. This is because the asynchrony in reproduction translates into asynchrony in the attainment of the overwintering developmental stage in the (diapausing) offspring generation (Fig. 7). Thus, early maturation (i.e., short development time) and high investment in early reproduction (i.e., high reproductive effort) are selected for in the direct development generation to ensure survival of their diapausing offspring. Interyear variation in season beginning date and season length is apparently needed to make selection for the production of the direct generation favoring short development time in the first (diapause) larval generation so strong that it outweighs the selection for high survival of the offspring of the direct generation favoring short development time in the second (direct) larval generation in a life cycle with egg diapause. With pupal or adult diapause, both selection pressures (induction of direct development, asynchronous reproduction in the direct generation) are favoring relatively fast development in the direct development generation, so no qualitative effect of environmental stochasticity on juvenile development is predicted.

Temporal variation in season length affects the evolution of alternative life histories and phenology. Relatively low variation in season length among years favors high investment in the direct generation and optimization of alternative life history phenotypes in relation to the average season. When uncertainty in season length increases, there is a threshold where a switch to a bet‐hedging type of life history and phenology takes place. At the threshold, the expression of the diapause phenotype sharply increases, resulting in univoltine phenology beyond the threshold if the mean season length inflicts strong time constraints for bivoltine phenology (Figs. 3, 4), and in partially bivoltine phenology if the time constraints are relaxed (Appendix S3). Variance in season length generates exaggerated variance in fitness in bivoltine populations because, depending on the season length, reproductive success of the direct generation may vary from complete failure to high success. On the other hand, diapausing offspring produced by the diapause generation generally experience relaxed time constraints in (partially and completely) bivoltine populations and thus have a high survival probability even in exceptionally short seasons. Hence, increasing expression of the diapause phenotype decreases variance in fitness, but at the cost of reduced arithmetic mean fitness, which perfectly fits in the definition of bet‐hedging (Seger and Brockmann 1987; Starrfelt and Kokko 2012). Our result of a threshold in temporal heterogeneity, beyond which bet‐hedging is favored agrees with genetic models predicting a similar threshold (Slatkin and Lande 1976; Bull 1987; Sasaki and Ellner 1995; Scheiner 2014). Also, Roff's (1983) analysis suggests a threshold‐type switch to plastic development time adjustment with increasing uncertainty in season length. On the grounds of the present and the above‐mentioned earlier work, a threshold‐type of switch to bet‐hedging rather than a gradual change may be generally expected with increasing temporal uncertainty in the environment.

Within‐season mortality risk due to low temperatures had a minimal effect on life history differentiation between alternative developmental pathways, but clearly affected the predicted pathway‐specific absolute trait values especially in the diapause pathway when mean season length was short (see Appendix S3). This is because within‐season mortality effectively imposes time constraints on the life cycle, so its implications on life history evolution would become exaggerated when mean season length is relatively short.

The life history clines predicted to evolve along a gradient of mean season length with temporal variation (Figs. 5, 6) are qualitatively in accordance with corresponding predictions for deterministic environments (Roff 1980; Kivelä et al. 2009, 2013), except for reproductive effort and adult life span. In general, the predicted clines conform to the saw‐tooth pattern with monotonic change with constant voltinism, interrupted by an abrupt change in the opposite direction when voltinism changes. Increasing temporal variation in season length induced a qualitative change in the cline for reproductive effort. The direction of change in the diapause pathway at the change from univoltine to bivoltine phenology was reversed with high temporal variation in season length. In the direct development pathway, reproductive effort showed similar clinal variation independently of temporal variation in season length. Consequently, the degree of divergence in reproductive effort between the diapause and direct development pathways exaggerates in time constrained bivoltine populations with a high uncertainty in season length, although the direction of divergence remains unaffected. Decreasing reproductive effort in the diapause pathway translates into an extended reproductive life span in the adult stage, which facilitates the production of phenotypically diverse offspring (i.e., bet‐hedging) as early‐life reproduction tends to produce directly developing offspring and late‐life reproduction diapausing offspring. This is reflected in the cline of adult life span in the diapause pathway as adult life span continues to lengthen toward increasing mean season length without a saw‐tooth change at changing voltinism. The direct generation experiences, on average, similar and severe time constraints independently of temporal variation in season length, if bivoltine phenology is time constrained. Therefore, reproductive effort is predicted to be mainly affected by the mean season length in the direct development pathway, which also results in a relatively short adult life span associated with the direct development. When time constraints for bivoltine phenology relax, the reproductive efforts in the diapause and direct development pathways converge even if season length is uncertain, and a sharp shortening of the adult life span occurs in the diapause pathway. Empirical data are consistent with the predicted clinal variation in juvenile traits (reviewed in Kivelä et al. 2013), but clinal variation in the adult traits that we investigated here has not been rigorously studied so far.

We assumed density‐independent selection in our analysis. Relaxing this assumption by adding density dependency of fitness would probably increase the expression of the diapause phenotype compared to the present predictions and thus affect voltinism shifts. This is implied by the general tendency of a high predicted dormancy frequency in density‐dependent models compared to density‐independent ones (Bulmer 1984; Ellner 1985; Rajon et al. 2014). However, the effect of density dependency on the current predictions needs to be rigorously analyzed, and future studies should address this issue.

In conclusion, our analysis demonstrates that environmental uncertainty favors adaptive life history differentiation between diapausing and directly developing insects in seasonal environments, which significantly adds realism to insect life history theory. As another novel aspect, life cycle variation is predicted to have a stronger effect on the evolution of alternative life histories in stochastic (this study) than in deterministic environments (Kivelä et al. 2013) because time constraints become effectively stronger and more life cycle specific in the former case. Yet, current empirical data solely include species with pupal or adult diapause, which does not allow us to assess the biological relevance of the prediction. Seasonality as such favors adaptive predictive plasticity resulting in polyphenic expression of life histories (Kivelä et al. 2013; this study). Increasing stochasticity in seasonality, in turn, favors bet‐hedging in the temporal pattern how alternative phenotypes are expressed (Halkett et al. 2004; this study). This is then reflected in the trait values expressed under the alternative developmental pathways (this study). Increasing variation in season length induces a switch toward decreasing investment in the directly developing generation to reduce variance in fitness (cf. Seger and Brockmann 1987; Starrfelt and Kokko 2012). More generally, this implies that polyphenisms emerging via predictive plasticity in relation to deterministic environmental variation may facilitate the evolution of diversified bet‐hedging strategies (sensu Starrfelt and Kokko 2012) under environmental stochasticity. We also see no sharp distinction between predictive plasticity and diversified bet‐hedging because a significant adaptive component of phenotype expression remains despite some temporal overlap in the expression of the alternative phenotypes. Provided that selection is density‐independent and seasonality is included in the analysis, it appears possible to derive qualitatively realistic predictions whether a deterministic or stochastic environment is assumed (Kivelä et al. 2013; this study), which validates earlier theory (Roff 1980; Iwasa et al. 1994; Abrams et al. 1996; Gotthard et al. 2007; Kivelä et al. 2009, 2013) and explains the good fit between theoretical predictions and empirical evidence (reviewed in Kivelä et al. 2013).

## Conflict of Interest

None declared.

## Data Accessibility

R‐code of the model is available as Appendices S1 and S2.

## Supporting information


**Figure S1.** Locations (black points) where climate data was obtained.
**Figure S2.** Probability of a frost occurring within a season in relation to time since the beginning of the season with different values of *τ* when season length is 40 days.

**Appendix S1.** R‐code of the analysis: fixed mean season length.

**Appendix S2.** R‐code of the analysis: clinal variation.

**Appendix S3.** Results and Discussion.

## Appendix

The model is based on the deterministic model by Kivelä et al. (2013). Growth is modeled with the power function model (Tammaru and Esperk 2007), pupal mass, *m*_pupa_, being

(A1) $$m_{\mathrm{pupa}}=ct_{\mathrm{larva}}^{B},$$

where *c* is larval growth rate, *t*_larva_ is duration of the larval period, and *B *=* *1/(1 − *g*), *g* being an allometric exponent relating anabolism to body mass (Tammaru and Esperk 2007). The minimum mass required for a successful metamorphosis into the adult stage is set as *m*_min_ (i.e., we require that *m*_pupa_ ≥ *m*_min_). According to Kivelä et al. (2013), pupal mass is assumed to determine potential maximum daily fecundity, *f*_max_, of adult females so that

(A2) $$f_{\max}=a\left(1-e^{-{\mathrm{bm}}_{\mathrm{pupa}}}\right),$$

where *a* defines an asymptote for *f*_max_ and *b* determines the rate of approaching the asymptote with increasing *m*_pupa_. Then, the expected fecundity at age *i*,* F(i,E)*, can be expressed as a function of reproductive effort, *E* (0 ≤ *E *≤* *1), as

(A3) $$F\left(i,E\right)=f_{\max}\left(1-e^{-c_{1}E}\right){\left(1-E^{c_{2}}\right)}^{i-1},$$

where *c*_1_ and *c*_*2*_ are parameters and *i = 1, …, ω*,* ω* being adult lifespan, defined so that *F(i,E)* < 0.1 when *i *> *ω*. Reproductive effort is the proportion of energy allocated to reproduction (assumed constant over life span), which results in the above‐defined trade‐off between reproductive effort and adult life span. Specifically, values of *c*_*1*_ and *c*_*2*_ (Table 1) that specify fecundity to be an increasing concave function of reproductive effort and survival probability to be a decreasing concave function of reproductive effort are used (Kivelä et al. 2009; see Schaffer 1974b for justification).

Extrinsic larval (*M*_juv_) and adult (*M*_ad_) mortality rates are included in the model, larval mortality rate increasing with increasing growth rate. Larval mortality rate is, thus, given by

(A4) $$M_{\mathrm{juv}}\left(c\right)=M_{\mathrm{juv}0}+\frac{dc^{k}}{c_{d}^{k}},$$

where *M*_juv0_ is growth rate‐independent component of larval mortality rate, *c*_d_ and *d* define the growth rate‐dependent component of *M*_juv_ (i.e., *M*_juv_ *(c*_d_ *)* = *M*_juv0_ **+ *d*), and *k* determines the curvature of the function *M*_juv_ *(c)*. Taking into account larval and adult mortality, it follows from the above derivations that the expected lifetime offspring production (net reproductive rate), *R*_0(Dp)_, in the diapause pathway is given by

(A5) $$\begin{array}{cc} R_{0(\mathrm{Dp})}=\;\; & e^{-M_{\mathrm{juv}}\left(c_{\mathrm{Dp}}\right)t_{\mathrm{larva}(\mathrm{Dp})}}\sum_{i=1}^{\omega_{\mathrm{Dp}}}e^{-M_{\mathrm{ad}}i}f_{\max}\left(1-e^{-c_{1}E_{\mathrm{Dp}}}\right)\\ & {\left(1-E_{\mathrm{Dp}}^{c_{2}}\right)}^{i-1}, \end{array}$$

where the subscript “Dp” refers to the diapause pathway. The direct development pathway is referred to the subscript “Di”. If a direct generation emerges, the expected number of eggs laid by all the direct generation females on day *j* (*j *=* *1, …, *ω*_Di_ − 1 +  *n*_coh_; *n*_coh_ is the number of cohorts that develop directly into adults, which depends on *t*_larva(Di)_, *D** and *ω*_Dp_) since the emergence of the first direct generation females, *F*_Di_(*j*,*E*_Di_), is

(A6) $$\begin{array}{cc} F_{\mathrm{Di}}\left(j,E_{\mathrm{Di}}\right) & =e^{-M_{\mathrm{juv}}\left(c_{\mathrm{Di}}\right)t_{larva(\mathrm{Di})}}\sum_{x=1}^{n_{\mathrm{coh}}}N_{x}e^{-M_{\mathrm{ad}}\left(j-x+1\right)}f_{\max}\\ & \;\left(1-e^{-c_{1}E_{\mathrm{Di}}}\right){\left(1-E_{\mathrm{Di}}^{c_{2}}\right)}^{j-x}, \end{array}$$

where *N*_*x*_ is the number of adults in cohort *x*. The sum in equation (A6) is zero, if *j − x + *1 *<* 1, or if *j − x + *1 *>* *ω*_Di_. The expected lifetime offspring production for the direct development pathway life history (*R*_0(Di)_) is given by

(A7) $$\begin{array}{cc} R_{0(\mathrm{Di})}= & \;\;e^{-M_{juv}\left(c_{\mathrm{Di}}\right)t_{\mathrm{larva}(\mathrm{Di})}}\sum_{i=1}^{\omega_{\mathrm{Di}}}e^{-M_{\mathrm{ad}}i}f_{\max}\left(1-e^{-c_{1}E_{\mathrm{Di}}}\right)\\ & {\left(1-E_{\mathrm{Di}}^{c_{2}}\right)}^{i-1}. \end{array}$$

Because larval development time (*t*_larva(•)_) and reproductive effort (*E*_*•*_) were considered as independent traits, growth rate optimization was conducted for each analyzed combination of *t*_larva(•)_ and *E*_*•*_. A range of growth rates (*c*_*•*_) was analyzed in each *t*_larva(•)_−*E*_*•*_ combination. The minimum analyzed growth rate was the value of *c*_*•*_ that resulted in the minimum mass for a successful metamorphosis, *m*_min_, within the investigated *t*_larva(•)_. The maximum analyzed *c*_*•*_, *c*_*z•*_, defined as

(A8) $$c_{z∙}={\left[\frac{-\left(\ln\left(z\right)+M_{\mathrm{juv}0}t_{\mathrm{larva}(∙)}\right)c_{d}^{k}}{dt_{\mathrm{larva}(∙)}}\right]}^{\frac{1}{k}},$$

resulted in a probability of *z* to survive the larval stage (note that mortality risk increases with increasing *c*_*•*_). *R*_*0*(•)_ was calculated with 40 equally spaced values of *c*_*•*_ between the minimum and maximum defined above (including the endpoints), and the value of *c*_*•*_ that maximized *R*_*0*(•)_ was considered to be the *c*_*•*_ associated with the values of *t*_*larva*(•)_ and *E*_*•*_ under investigation.

The minimum analyzed *t*_larva(•)_ was the *t*_larva(•)_ (rounded to the nearest integer) that resulted in the attainment of *m*_min_ with the growth rate of *c*_*z*_ (equation (A8)) specific to that particular *t*_larva(•)_. The maximum *t*_larva(Dp)_ analyzed was *μ*_T_ − *t*_pupa_ − 1 (*μ*_T_ is the mean season length in the analyzed selection regime; *t*_pupa_ = 1), whereas the upper limit for *t*_larva(Di)_ was the longest *t*_larva(Di)_ facilitating the emergence of a direct generation. 67 values of *E*_*•*_ between 0.01 and 1 (including the endpoints; increment 0.015) were analyzed.
