# Supplementary material for: Evolution of alternative insect life histories in stochastic seasonal environments
Source: Ecol Evol. 2016 Jul 15;6(16):5596–613. doi: 10.1002/ece3.2310 (PMC4983577; doi:10.1002/ece3.2310)
Supplement: Supplementary file 1 — Figure S1. Locations (black points) where climate data was obtained. Figure S2. Probability of a frost occurring within a season in relation to time since the beginning of the season with different values of τ when season length is 40 days. [file ECE3-6-5596-s001.docx]

**Supplementary figures**

**Figure S1.** Locations (black points) where climate data was obtained.

**Figure S2.** Probability of a frost occurring within a season in relation to time since the beginning of the season with different values of *τ* when season length is 40 days.
