# Supplementary material for: Evolution of alternative insect life histories in stochastic seasonal environments
Source: Ecol Evol. 2016 Jul 15;6(16):5596–613. doi: 10.1002/ece3.2310 (PMC4983577; doi:10.1002/ece3.2310)
Supplement: Supplementary file 4 — Appendix S3. Results and Discussion. [file ECE3-6-5596-s004.docx]

**Appendix S3. Results and Discussion**

**Severe time constraints (*μ_T_* = 36 days)**

*Overview*

Although the pattern of variation in the relative (Figs 3 and 4) and absolute (see below) trait values is largely quite coarse, the fitness surfaces are very smooth (Fig A1). The coarse variation in the underlying traits is a consequence of the inclusion of both discrete (*D**, *t_larva(•)_* and *ω_•_*) and continuous (*c_•_*, *m_pupa(•)_*, *E_•_* and *R_0(•)_*) traits in the model.

The fitness benefit of bivoltinism is evident (Fig. A1; compare Fig. A1 to Figs 3 and 4), but in very variable environments bivoltinism becomes so risky that natural selection favours univoltinism there. Fitness increases with decreasing standard deviation of season length (i.e., increasing environmental stability) and decreasing risk of within-season frosts (i.e., increasing favourability of the environment) independently of the diapause strategy and phenology (Fig. A1), as would be intuitively expected. The proximate reason underlying the switch from univoltine to bivoltine phenology with increasing standard deviation of season length is a change in the critical date of diapause induction. The critical date becomes much earlier at the transition (Fig. A2). There is no variation in the critical date within univoltine phenology, and only very little within bivoltine phenology (Fig. A2).

Quantitative fitness comparisons between pupal and egg diapause strategies should be avoided, because the structure of our model generates some quantitative difference between them. This is because no mortality was assumed to take place during diapause, which means that all offspring of the direct generation survive until the end of season under egg diapause (no larval mortality before diapause) whereas only a proportion of $e^{-M_{juv}(c_{Dp})t_{larva(Dp)}}$ will survive until the end of the season with pupal diapause (whole larval stage under mortality risk before diapause). That explains the higher fitness for egg than for pupal diapause (Fig. A1).

**Figure A1. Geometric mean fitness with pupal (A) and egg (B) diapause** **in time-constrained bivoltine phenology** in relation to standard deviation of season length (*σ_T_*) and within-season risk of frosts (*τ*; risk increases with decreasing value of *τ*). Parameter values were: *c_1_* = 2.5, *c_2_* = 2, *a* = 50, *b* = 0.01, *c_d_* = 0.05, *k* = 2, *z* = 0.001, *B* = 3, *m_min_* = 80, *t_pupa_* = 1, *d* = 0.15, *M_juv0_* = 0.01, *M_ad_* = 0.15, *r* = 1 (A), *r* = 0 (B) and *μ_T_* = 36.

**Figure A2.** **Critical date** (parameter *D**; days since 31^st^ December) **that maximizes** **geometric mean fitness with pupal (A) and egg (B) diapause** **in time-constrained bivoltine phenology** in relation to standard deviation of season length (*σ_T_*) and within-season risk of frosts (*τ*; risk increases with decreasing value of *τ*). Lighter colour indicates later critical date. Dark grey indicates a region where a direct generation does not emerge. Parameter values were: *c_1_* = 2.5, *c_2_* = 2, *a* = 50, *b* = 0.01, *c_d_* = 0.05, *k* = 2, *z* = 0.001, *B* = 3, *m_min_* = 80, *t_pupa_* = 1, *d* = 0.15, *M_juv0_* = 0.01, *M_ad_* = 0.15, *r* = 1 (A), *r* = 0 (B) and *μ_T_* = 36.

*Developmental pathway-specific trait values*

The change from a bivoltine to a univoltine phenology due to increasing standard deviation of season length (*σ_T_*; see Figs 3 and 4) had a pronounced effect on the predicted diapause pathway trait values; larval development time and adult life span were much longer, larval growth rate and reproductive effort were much lower, body size was much larger and fecundity was much higher within that part of the parameter space where phenology was univoltine than where bivoltinism prevailed (Figs A3 and A4). These predictions were qualitatively insensitive to diapause strategy (egg vs. pupal diapause), and were expected as time constraints become relaxed when phenology changes from bi- to univoltinism, given that mean season length remains the same. Insects are, thus, predicted to take the advantage of the increased time available per generation by growing for a longer time, but at a slower rate, to attain a larger size, and thus higher fecundity. At the same time, investment to early reproduction at the expense of lifetime fecundity is predicted to decrease, as indicated by the decrease in reproductive effort and consequent extension in adult life span.

Within that part of the analysed parameter space where phenology was bivoltine, there was relatively little variation in the diapause pathway trait values that maximized geometric mean fitness (Figs A3 and A4). In environments with a lot of temporal variation in season length, univoltinism was predicted to evolve. Under univoltinism, where only the diapause pathway trait values are expressed, particularly the geometric mean fitness-maximizing values of larval development time, body size and fecundity changed towards the trait values predicted for the diapause pathway under bivoltine phenology when standard deviation of season length and within-season frost risk increased (*σ_T_* increased and *τ* decreased). These responses are understandable as the time constraints effectively become more severe when standard deviation of season length increases (the risk of very short seasons increases) and/or within-season frost risk increases (season length effectively becomes shorter).

In the direct development pathway, relatively little variation was predicted in the geometric mean fitness-maximizing trait values in most traits in relation to standard deviation of season length and within-season frost risk independently of diapause strategy (Figs A5 and A6). Body size was invariably the minimum required for successful metamorphosis (Figs A5C and A6C) like in the diapause pathway under bivoltinism (Figs A3C and A4C). With pupal diapause, direct development pathway reproductive effort was generally predicted to decrease with decreasing risk of within-season frosts (Fig. A5D), which is expected because decreasing within-season frost risk effectively lengthens the season, and so should decrease the intensity of selection for early reproduction. With egg diapause, the predicted variation in direct generation reproductive effort in relation to standard deviation of season length and within-season frost risk was more complex (and lacked a clear pattern) than that predicted with pupal diapause (compare Fig. A6D to Fig. A5D).

**Figure A3. Life history variation in the diapause pathway in time-constrained bivoltine phenology with pupal diapause.** The geometric mean-maximizing larval development time (days) (A), larval growth rate (mg day^-B^) (B), pupal mass (mg) (C), reproductive effort (D), adult life span (days) (E) and lifetime fecundity (F) in relation to standard deviation of season length (*σ_T_*) and within-season risk of frosts (*τ*; risk increases with decreasing value of *τ*). Note that the direction of the axes varies among plots, and the corner with the lowest values of *σ_T_* and *τ* is indicated by a black dot. Parameter values were: *c_1_* = 2.5, *c_2_* = 2, *a* = 50, *b* = 0.01, *c_d_* = 0.05, *k* = 2, *z* = 0.001, *B* = 3, *m_min_* = 80, *t_pupa_* = 1, *d* = 0.15, *M_juv0_* = 0.01, *M_ad_* = 0.15, *r* = 1 and *μ_T_* = 36.

**Figure A4. Life history variation in the diapause pathway in time-constrained bivoltine phenology with egg diapause.** The geometric mean-maximizing larval development time (days) (A), larval growth rate (mg day^-B^) (B), pupal mass (mg) (C), reproductive effort (D), adult life span (days) (E) and lifetime fecundity (F) in relation to standard deviation of season length (*σ_T_*) and within-season risk of frosts (*τ*; risk increases with decreasing value of *τ*). Note that the direction of the axes varies among plots, and the corner with the lowest values of *σ_T_* and *τ* is indicated by a black dot. Parameter values were: *c_1_* = 2.5, *c_2_* = 2, *a* = 50, *b* = 0.01, *c_d_* = 0.05, *k* = 2, *z* = 0.001, *B* = 3, *m_min_* = 80, *t_pupa_* = 1, *d* = 0.15, *M_juv0_* = 0.01, *M_ad_* = 0.15, *r* = 0 and *μ_T_* = 36.

**Figure A5. Life history variation in the direct development pathway in time-constrained bivoltine phenology with pupal diapause.** The geometric mean-maximizing larval development time (days) (A), larval growth rate (mg day^-B^) (B), pupal mass (mg) (C), reproductive effort (D), adult life span (days) (E) and lifetime fecundity (F) in relation to standard deviation of season length (*σ_T_*) and within-season risk of frosts (*τ*; risk increases with decreasing value of *τ*). Note that the direction of the axes varies among plots, and the corner with the lowest values of *σ_T_* and *τ* is indicated by a black dot. Parameter values were: *c_1_* = 2.5, *c_2_* = 2, *a* = 50, *b* = 0.01, *c_d_* = 0.05, *k* = 2, *z* = 0.001, *B* = 3, *m_min_* = 80, *t_pupa_* = 1, *d* = 0.15, *M_juv0_* = 0.01, *M_ad_* = 0.15, *r* = 1 and *μ_T_* = 36.

**Figure A6. Life history variation in the direct development pathway in time-constrained bivoltine phenology with egg diapause.** The geometric mean-maximizing larval development time (days) (A), larval growth rate (mg day^-B^) (B), pupal mass (mg) (C), reproductive effort (D), adult life span (days) (E) and lifetime fecundity (F) in relation to standard deviation of season length (*σ_T_*) and within-season risk of frosts (*τ*; risk increases with decreasing value of *τ*). Note that the direction of the axes varies among plots, and the corner with the lowest values of *σ_T_* and *τ* is indicated by a black dot. Parameter values were: *c_1_* = 2.5, *c_2_* = 2, *a* = 50, *b* = 0.01, *c_d_* = 0.05, *k* = 2, *z* = 0.001, *B* = 3, *m_min_* = 80, *t_pupa_* = 1, *d* = 0.15, *M_juv0_* = 0.01, *M_ad_* = 0.15, *r* = 0 and *μ_T_* = 36.

**Relaxed time constraints (*μ_T_* = 50 days)**

*Overview*

With relaxed time constraints for bivoltine phenology, bivoltinism was predicted to evolve within the whole analysed parameter space. Bivoltinism emerged even if we included more variable selection regimes (maximum *σ_T_* was 8 days with *μ_T_* = 50 days) in this analysis than in the corresponding time-constrained analysis (maximum *σ_T_* was 6 days with *μ_T_* = 36 days).

Geometric mean fitness varied in relation to standard deviation of season length and within-season frost risk very similarly as in the time-constrained analysis; fitness increased towards increasing stability (i.e., decreasing *σ_T_*) and favourability (i.e., increasing *τ*) of the environment (compare Fig. A7 to Fig. A1). There was also a rather steep decline in fitness with increasing standard deviation of season length between standard deviations of 4 and 5 days (Fig. A7A, B), which was not a consequence of changing voltinism but a steep decrease in the average size of the directly developing generation (Fig. A7C, D). Simultaneously with the decreasing average size of the direct generation (i.e., fewer offspring cohorts produced by the diapause generation enter direct development, on average), the geometric mean fitness-maximizing reproductive effort and adult life span in the diapause pathway tended to change towards values expected under a univoltine phenology (see below), while the other traits changed as would be expected under intensifying time constraints. Overall, this change implies that increasing environmental variation at long mean seasons favours the evolution of a partially bivoltine phenology where, on average, only the earliest offspring cohorts of the diapause generation enter direct development. Diapause pathway trait values that approach those expected under a univoltine phenology or under severe time constraints, together with the decreasing average size of the direct generation, mean that an increasing proportion of offspring produced by the diapause generation enter diapause and escape the risk of complete failure of the direct generation in exceptionally short seasons. Accordingly, natural selection seemingly favours a bet-hedging strategy when mean season length is highly variable but relatively long; univoltinism ensures some fitness, but the benefits of bivoltinism is taken in years with exceptionally long seasons. Some potential fecundity is sacrificed to reach early maturation, which is clearly advantageous under the risk of short seasons – and, on the other hand, facilitates the emergence of a direct generation in favourable years. The proximate mechanism underlying this kind of bet-hedging is earlier critical date of diapause induction (compare Fig. A8 to Fig. A7). The switch to an earlier critical date is sudden and not gradual (Fig. A8), critical date becoming 12–18 days earlier at the transition.

**Figure A7.** **Geometric mean fitness (A, B) and average proportion of diapause generation offspring entering direct development (C, D) with pupal (A, C) and egg (B, D) diapause** **in bivoltine phenology with relaxed time constraints** in relation to standard deviation of season length (*σ_T_*) and within-season risk of frosts (*τ*; risk increases with decreasing value of *τ*). Parameter values were: *c_1_* = 2.5, *c_2_* = 2, *a* = 50, *b* = 0.01, *c_d_* = 0.05, *k* = 2, *z* = 0.001, *B* = 3, *m_min_* = 80, *t_pupa_* = 1, *d* = 0.15, *M_juv0_* = 0.01, *M_ad_* = 0.15, *r* = 1 (A, C), *r* = 0 (B, D) and *μ_T_* = 50.

**Figure A8.** **Critical date** (parameter *D**; days since 31^st^ December) **that maximizes geometric mean fitness with pupal (A) and egg (B) diapause** **in bivoltine phenology with relaxed time constraints** in relation to standard deviation of season length (*σ_T_*) and within-season risk of frosts (*τ*; risk increases with decreasing value of *τ*). Lighter colour indicates later critical date (note that a particular colour indicates different trait values in A and B). Parameter values were: *c_1_* = 2.5, *c_2_* = 2, *a* = 50, *b* = 0.01, *c_d_* = 0.05, *k* = 2, *z* = 0.001, *B* = 3, *m_min_* = 80, *t_pupa_* = 1, *d* = 0.15, *M_juv0_* = 0.01, *M_ad_* = 0.15, *r* = 1 (A), *r* = 0 (B) and *μ_T_* = 50.

*Trait differentiation between the alternative developmental pathways*

A qualitatively similar life cycle effect on the direction of trait differentiation between the alternative developmental pathways was predicted under relaxed time constraints for bivoltine phenology as under severe time constraints (compare Figs A9 and A10 to Figs 3 and 4). Hence, the geometric mean fitness-maximizing values of larval development time and adult life span tended to be shorter, growth rate higher and fecundity lower under direct than under diapause development with pupal diapause strategy (Fig. A9). With egg diapause, these inter-pathway differences tended to reverse, but equal trait values in the alternative developmental pathways were also predicted within a large part of the analysed parameter space (Fig. A10). The reversal of the direction of trait differentiation with egg diapause was less evident for adult life span than it was in the severely time-constrained case. Indeed, a shorter life span under direct development than under diapause was more often predicted than the opposite. Contrary to the predictions for severely time-constrained bivoltinism, inter-pathway differentiation in body size was also predicted with relaxed time-constraints so that directly developing individuals would be smaller than diapausing ones with pupal diapause, whereas the reverse or equality of body sizes was more often expected with egg diapause (Figs A9 and A10). For reproductive effort, equal trait values were mainly predicted with low standard deviations of season length (bivoltine phenology). At the transition to a partially bivoltine phenology and beyond it (i.e., towards higher standard deviations of season length), higher reproductive effort was invariably predicted for the direct development pathway than for the diapause pathway independently of life cycle (Figs A9D and A10D). The change from a bivoltine to a partially bivoltine phenology is evident also in the predictions for adult life span as a relatively short life span was invariably predicted for direct development under a partially bivoltine phenology (Figs A9E and A10E). This represents a correlated response to the predicted shift in reproductive effort. The change from a bivoltine to a partially bivoltine phenology has less effect on the predicted trait differentiation between the developmental pathways in the other traits.

**Figure A9.** **Contour diagrams of relative life history differences between the direct development and the diapause pathway in bivoltine phenology with relaxed time constraints with pupal diapause.** Trait value in the direct development pathway is divided by the trait value in the diapause pathway. The ratio is shown for larval development time (A), larval growth rate (B), pupal mass (C), reproductive effort (D), adult life span (E) and lifetime fecundity (F) in relation to standard deviation of season length (*σ_T_*) and within-season risk of frosts (*τ*; risk increases with decreasing value of *τ*). Ratios less than one are indicated by blue and ratios higher than one by red. Darker colour indicates increasing differentiation between the developmental pathways (note that a particular darkness of blue or red indicates different trait differentiation in different panels), and a ratio of one (i.e., equality of life histories) is indicated by white colour. Parameter values were: *c_1_* = 2.5, *c_2_* = 2, *a* = 50, *b* = 0.01, *c_d_* = 0.05, *k* = 2, *z* = 0.001, *B* = 3, *m_min_* = 80, *t_pupa_* = 1, *d* = 0.15, *M_juv0_* = 0.01, *M_ad_* = 0.15, *r* = 1, *μ_T_* = 50, *μ_I_* = 155 and *σ_I_* = 3.2.

**Figure A10. Contour diagrams of relative life history differences between the direct development and the diapause pathway in bivoltine phenology with relaxed time constraints with egg diapause.** Trait value in the direct development pathway is divided by the trait value in the diapause pathway. The ratio is shown for larval development time (A), larval growth rate (B), pupal mass (C), reproductive effort (D), adult life span (E) and lifetime fecundity (F) in relation to standard deviation of season length (*σ_T_*) and within-season risk of frosts (*τ*; risk increases with decreasing value of *τ*). See Figure A9 for explanation of the colours. Parameter values were: *c_1_* = 2.5, *c_2_* = 2, *a* = 50, *b* = 0.01, *c_d_* = 0.05, *k* = 2, *z* = 0.001, *B* = 3, *m_min_* = 80, *t_pupa_* = 1, *d* = 0.15, *M_juv0_* = 0.01, *M_ad_* = 0.15, *r* = 0, *μ_T_* = 50, *μ_I_* = 155 and *σ_I_* = 3.2.

*Pathway-specific trait values*

The change from a bivoltine to a partially bivoltine phenology had a clear effect on diapause pathway trait values, especially with pupal diapause (Figs A11 and A12). With pupal diapause, the geometric mean fitness-maximizing larval development time shortened, growth rate increased, body size become smaller, reproductive effort decreased, adult life span lengthened and fecundity tended to decrease at the transition (Fig. A11). With egg diapause, diapause pathway reproductive effort and adult life span were predicted to respond similarly to the shift in voltinism as with pupal diapause (Fig. A12D, E), whereas the geometric mean fitness-maximizing values of the other traits fluctuated with respect to both standard deviation of season length and within-season frost risk without a clear pattern (Fig. A12A, B, C, F).

In the direct development pathway, the geometric mean fitness-maximizing trait values were not only affected by standard deviation of season length but, to some extent, also by within-season frost risk independently of diapause strategy (Figs A13 and A14). With pupal diapause, the change from a bivoltine to a partially bivoltine phenology clearly reflected to direct generation trait values only for decreasing reproductive effort (Fig. A13D), and for prolonged adult life span (Fig. A13E). With egg diapause, variation in the geometric mean fitness-maximizing trait values lacked a clear pattern (Fig. A14). However, the shortest development times, highest growth rates, smallest body sizes and lowest fecundities tended to be associated with a high within-season frost risk independently of diapause strategy (Figs A13 and A14). This implies that increasing within-season frost risk (decreasing *τ*) increases the effective time constraints for the direct generation to the extent that affects the evolution of trait values under direct development even when the mean season length is long and the frost risk level is overall lower than in environments where seasons are, on average, shorter.

**Figure A11. Life history variation in the diapause pathway in bivoltine phenology with relaxed time constraints with pupal diapause.** The geometric mean-maximizing larval development time (days) (A), larval growth rate (mg day^-B^) (B), pupal mass (mg) (C), reproductive effort (D), adult life span (days) (E) and lifetime fecundity (F) in relation to standard deviation of season length (*σ_T_*) and within-season risk of frosts (*τ*; risk increases with decreasing value of *τ*). Note that the direction of the axes varies among plots, and the corner with the lowest values of *σ_T_* and *τ* is indicated by a black dot. Parameter values were: *c_1_* = 2.5, *c_2_* = 2, *a* = 50, *b* = 0.01, *c_d_* = 0.05, *k* = 2, *z* = 0.001, *B* = 3, *m_min_* = 80, *t_pupa_* = 1, *d* = 0.15, *M_juv0_* = 0.01, *M_ad_* = 0.15, *r* = 1 and *μ_T_* = 50.

**Figure A12. Life history variation in the diapause pathway in bivoltine phenology with relaxed time constraints with egg diapause.** The geometric mean-maximizing larval development time (days) (A), larval growth rate (mg day^-B^) (B), pupal mass (mg) (C), reproductive effort (D), adult life span (days) (E) and lifetime fecundity (F) in relation to standard deviation of season length (*σ_T_*) and within-season risk of frosts (*τ*; risk increases with decreasing value of *τ*). Note that the direction of the axes varies among plots, and the corner with the lowest values of *σ_T_* and *τ* is indicated by a black dot. Parameter values were: *c_1_* = 2.5, *c_2_* = 2, *a* = 50, *b* = 0.01, *c_d_* = 0.05, *k* = 2, *z* = 0.001, *B* = 3, *m_min_* = 80, *t_pupa_* = 1, *d* = 0.15, *M_juv0_* = 0.01, *M_ad_* = 0.15, *r* = 0 and *μ_T_* = 50.

**Figure A13. Life history variation in the direct development pathway in bivoltine phenology with relaxed time constraints with pupal diapause.** The geometric mean-maximizing larval development time (days) (A), larval growth rate (mg day^-B^) (B), pupal mass (mg) (C), reproductive effort (D), adult life span (days) (E) and lifetime fecundity (F) in relation to standard deviation of season length (*σ_T_*) and within-season risk of frosts (*τ*; risk increases with decreasing value of *τ*). Note that the direction of the axes varies among plots, and the corner with the lowest values of *σ_T_* and *τ* is indicated by a black dot. Parameter values were: *c_1_* = 2.5, *c_2_* = 2, *a* = 50, *b* = 0.01, *c_d_* = 0.05, *k* = 2, *z* = 0.001, *B* = 3, *m_min_* = 80, *t_pupa_* = 1, *d* = 0.15, *M_juv0_* = 0.01, *M_ad_* = 0.15, *r* = 1 and *μ_T_* = 50.

**Figure A14. Life history variation in the direct development pathway in bivoltine phenology with relaxed time constraints with egg diapause.** The geometric mean-maximizing larval development time (days) (A), larval growth rate (mg day^-B^) (B), pupal mass (mg) (C), reproductive effort (D), adult life span (days) (E) and lifetime fecundity (F) in relation to standard deviation of season length (*σ_T_*) and within-season risk of frosts (*τ*; risk increases with decreasing value of *τ*). Note that the direction of the axes varies among plots, and the corner with the lowest values of *σ_T_* and *τ* is indicated by a black dot. Parameter values were: *c_1_* = 2.5, *c_2_* = 2, *a* = 50, *b* = 0.01, *c_d_* = 0.05, *k* = 2, *z* = 0.001, *B* = 3, *m_min_* = 80, *t_pupa_* = 1, *d* = 0.15, *M_juv0_* = 0.01, *M_ad_* = 0.15, *r* = 0 and *μ_T_* = 50.
